# What Language Disorders Reveal About the Mechanisms of Morphological Processing

**DOI:** 10.3389/fpsyg.2021.701802

**Published:** 2021-11-29

**Authors:** Christina Manouilidou, Michaela Nerantzini, Brianne M. Chiappetta, M. Marsel Mesulam, Cynthia K. Thompson

**Affiliations:** ^1^Department of Comparative and General Linguistics, University of Ljubljana, Ljubljana, Slovenia; ^2^Department of Philology, University of Ioannina, Ioannina, Greece; ^3^Department of Communication Sciences and Disorders, Northwestern University, Evanston, IL, United States; ^4^Mesulam Center for Cognitive Neurology and Alzheimer’s Disease, Northwestern University, Chicago, IL, United States; ^5^Department of Neurology, Northwestern University, Chicago, IL, United States

**Keywords:** derivational morphology, morphological processing, pseudowords, primary progressive aphasia, stroke-induced aphasia, agrammatism

## Abstract

We addressed an understudied topic in the literature of language disorders, that is, processing of derivational morphology, a domain which requires integration of semantic and syntactic knowledge. Current psycholinguistic literature suggests that word processing involves morpheme recognition, which occurs immediately upon encountering a complex word. Subsequent processes take place in order to interpret the combination of stem and affix. We investigated the abilities of individuals with agrammatic (PPA-G) and logopenic (PPA-L) variants of primary progressive aphasia (PPA) and individuals with stroke-induced agrammatic aphasia (StrAg) to process pseudowords which violate either the syntactic (word class) rules (*reheavy) or the semantic compatibility (argument structure specifications of the base form) rules (*reswim). To this end, we quantified aspects of word knowledge and explored how the distinct deficits of the populations under investigation affect their performance. Thirty brain-damaged individuals and 10 healthy controls participated in a lexical decision task. We hypothesized that the two agrammatic groups (PPA-G and StrAg) would have difficulties detecting syntactic violations, while no difficulties were expected for PPA-L. Accuracy and Reaction Time (RT) patterns indicated: the PPA-L group made fewer errors but yielded slower RTs compared to the two agrammatic groups which did not differ from one another. Accuracy rates suggest that individuals with PPA-L distinguish *reheavy from *reswim, reflecting access to and differential processing of syntactic vs. semantic violations. In contrast, the two agrammatic groups do not distinguish between *reheavy and *reswim. The lack of difference stems from a particularly impaired performance in detecting syntactic violations, as they were equally unsuccessful at detecting *reheavy and *reswim. Reduced grammatical abilities assessed through language measures are a significant predictor for this performance, suggesting that the “hardware” to process syntactic information is impaired. Therefore, they can only judge violations semantically where both *reheavy and *reswim fail to pass as semantically ill-formed. This finding further suggests that impaired grammatical knowledge can affect word level processing as well. Results are in line with the psycholinguistic literature which postulates the existence of various stages in accessing complex pseudowords, highlighting the contribution of syntactic/grammatical knowledge. Further, it points to the worth of studying impaired language performance for informing normal language processes.

## Introduction

### Morphological Processing in Healthy Adults

An important dimension of word knowledge which has been found to affect lexical processing is *morphological structure.* Morphology changes a word’s form either to denote some grammatical function, e.g., *boy* > *boys* (singular > plural) or to create new lexical items with related (most of the time) meanings, e.g., *boy* > *boyish, boy* > *boyfriend.* The first operation is referred to as *inflection* (*boy-s*) while the other two are known as *derivation* (*boy-ish*) and *compounding* (*boy-friend*). In case of *inflection* and *derivation*, an *inflectional* or *derivational* morpheme attaches to a lexical stem, *boy* + -*s, boy* + *-ish* while in *compounding* two lexical stems merge together, *boy* + *friend.* These are highly productive operations in many languages.

The effects of complex structure on word processing have been studied extensively (see Amenta and Crepaldi, 2012 for a review). Till recently, the main issue among them was how morphologically complex words are accessed and how they are stored in the Mental Lexicon. In other words, the major question was whether we need to decompose them into their parts to access their meaning, e.g., *boy* + *ish* or whether we access them as one unit, e.g., *boyish.* This debate has had, and it still has proponents on both sides. Some researchers have claimed that morphological structure plays no role and that morphologically complex words are fully listed in the memory (Butterworth, 1983; Lukatela et al., 1987). Connectionist models are also against the representation of morphological structure in the mental lexicon (Elman et al., 1996; Sereno and Jongman, 1997). In contrast, other researchers have argued that complex words are obligatorily decomposed into their constituents and that the mental lexicon comprises only stems and affixes and not affixed words (Taft and Forster, 1975; Taft, 1988). Finally, several models of morphological processing have combined whole word access with affix-stripping, suggesting dual route processing for complex words (Frauenfelder and Schreuder, 1991; Chialant and Caramazza, 1995).

In the current literature, this remains an open debate, with recent papers providing evidence for both directions. This is certainly an important issue, nonetheless, its thorough discussion is beyond the scope of this paper. In our view, the balance might be turning in favor of decomposition route, as most recent neuroimaging studies suggests. Indeed, besides the numerous behavioral studies, a variety of neuroimaging studies from unimpaired populations about complex word processing have provided converging evidence that the human language processor has immediate access to constituent morphemes (see Münte et al., 1999; Lehtonen et al., 2011; Royle et al., 2012; Fruchter et al., 2013; Fruchter and Marantz, 2015; Leminen et al., 2019 for a review of neuroimaging data; Diependaele et al., 2009; Dominguez et al., 2010; Rastle and Davis, 2008 for a review of behavioral studies). This appears to be a process which operates in an early, automatic and semantic-blind way in both prefixed and suffixed words. Thus, in healthy populations, morphological decomposition takes place immediately after word viewing, resulting in the activation of both stem and affix, e.g., *teach* + *er*, for *teacher*.

If we assume that this is a property of the human language processing system, then it should be universal and it should operate independently of modality, i.e., visual vs. auditory. Lexical access takes place when sensory information is matched to lexical information. In auditory lexical access what activates lexical information is the first few phonemes (regardless of syllable structure), whereas in visual lexical access is the first (orthographically defined) syllable (Taft, 2004). Each modality is subject to additional restrictions related to the physical properties of input. For instance, in visual lexical decision factors such as frequency (Balota et al., 2004), family size (Bertram et al., 2000), derivational family entropy (del Prado Martín et al., 2004) facilitate lexical access. Similarly, in a relevant study about auditory recognition of prefixed words, Wurm et al. (2006) showed that cohort entropies, conditional root uniqueness points and morphological family size influenced lexical access of prefixed words. A general finding is that participants usually respond faster in visual lexical access compared to auditory but, importantly, there is no qualitative difference between their responses both behaviorally and in terms of EEG (Zunini et al., 2020) and MEG components (Brennan et al., 2014) which is suggestive of common underlying and universal ways of dealing with complex lexical items.

A big bulk of research regarding lexical access of complex words comes from pseudowords This spans from the early days of psycholinguistics (Caramazza et al., 1988; Laudanna et al., 1992; Burani et al., 1999) to the era of neuroimaging (Leinonen et al., 2009; Leminen et al., 2013; Kim et al., 2015) and it covers both auditory and visual lexical access. The reason for this choice is because pseudowords are devoid of lexical representations, and therefore, are supposed to be accessed through decomposition into their constituents. Furthermore, pseudowords are particularly useful for the exploration of prelexical effects of morphological segmentation, without lexical interference from the whole word. The main contribution of studies using pseudowords is that they robustly support multi-stage processing models for morphologically complex lexical items (such as the one adopted for the current study) ranging from behavioral experimental and modeling studies to neural evidence (for a review see Ripamonti et al., 2015). The use of pseudowords has also drawn criticism, mostly on the assumption that pseudowords lack semantics and that they are detached from the mental lexicon (Chuang et al., 2021). As we will see later, these lines of criticism are not directly relevant for the current study, as we do not treat pseudowords as meaningless units.

Several questions remain unsolved, however, pertaining to what happens once we have decomposed a complex word or pseudoword. Schreuder and Baayen (1995) in their meta-model which is designed to account for both visual and auditory processing of complex words^1^ described a two-stage post–decomposition process, consisting of *licensing*, during which each activated morpheme is validated through its subcategorization specifications (syntactic checking) and *composition*, during which we check whether the lexical representation of the whole word can be computed based on the semantic representations of the activated morphemes. In other words, syntactic licensing checks whether we are allowed to combine *teach* + *er* in terms of their syntactic properties and composition checks whether it makes sense to combine *teach* + *-er* on semantic grounds. With this in mind, we can postulate that all formations that do not respect either syntactic or semantic restrictions will fail to be recognized as real words, and their rejection will take place at different stage and timeframe.

Based on this and considering the latest advancements in lexical processing we can outline the architecture of complex pseudoword recognition. This would include a first stage, where obligatory decomposition occurs, and all lexicalized substrings are exposed. It is during this stage that pure non-words with the form of *stem* (non-existing) + *affix*, such as *pearn-able are rejected. The second stage includes *syntactic licensing*, during which all formations which violate the syntactic specifications of the base (grammatical class), are processed, and rejected. It is in this stage that a pseudoword of the type of *inappropriate stem* + *affix*, such as *river-able would be rejected. The third stage includes *semantic composition*, where formations such as *danceable would be rejected. Although both the *stem* (*dance*) and the *affix* (-*able*) are already activated in stage 2, it is not until this stage that semantic processing occurs, and participants decide on the well-formedness of semantic violations.

This architectural model has been confirmed in a variety of behavioral and neuroimaging studies, by using data from various languages and by employing either the violation paradigm described above which distinguishes between syntactic and semantic information or existing words. Several studies have investigated the temporal and spatial dynamics of grammatical category (*licensing*), showing that information associated with the syntactic category elicit an early left anterior negative ERP component (ELAN) peaking at about 250 ms after stimulus presentation (e.g., Hahne and Friederici, 1999; Hahne and Jescheniak, 2001). This response is identified usually at the inferior portion of the superior temporal gyrus. Data from MEG (Dikker et al., 2009; Linzen et al., 2013) confirm the early processing of grammatical category. Similarly, a separate body of studies advocate the existence of semantic composition as taking place at a later stage and at distinct brain-areas. Fruchter and Marantz (2015) were the first ones to establish the distinction between *lexeme lookup* and *semantic composition* by using derivational family entropy targeting *lexeme lookup* and a variable they called *derived semantic coherence* targeting *semantic composition.* The first variable elicited early activation (between 241 and 387 ms) in middle temporal gyrus, while the second elicited activation in orbitofrontal cortex at a later stage (between 431 and 500 ms). Finally, Whiting et al. (2014) compared complex words such as *teacher* to pseudo-complex words such as *corner* in a MEG experiment. While at early stages both types of words evoked same type of responses (inferior temporal gyrus and fusiform between 150 and 230 ms after stimulus presentation), there was greater activation for the pseudo-derived words at a later stage (between 300 and 360 ms) in the middle temporal gyrus. This later effect was interpreted by the authors as *lexicality effect* which amounts to semantic composition of an initially erroneously decomposed item.

The effects of *syntactic licensing* and *semantic composition* within one single experiment were first addressed by Manouilidou (2006, 2007) in a series of experiments. For instance, Manouilidou (2006, 2007) tested the ability of Greek-speaking individuals to detect violations of word formation with the aim to detect what kind of information is available after initial decomposition and morpheme recognition. The ultimate goal was to tease apart the contribution of *syntactic* and *semantic* information in deverbal structures. A variety of suffixes creating deverbal formations, nouns, and adjectives, was used. For instance, by using the Greek suffix -*tis* (equivalent to the English *-er*) which creates agentive nominalizations such as *pezo ‘play’* > *pex-tis ‘player’* we created syntactic violations, such as *potiri-tis (Noun + -*tis*) ‘glass-er’, and also semantic violations not respecting the argument structure specifications of the base such as *diaferistis ‘differ-er.’ The main finding of these behavioral experiments was that participants were faster and more accurate in detecting syntactic violations (*potiritis ‘glasser’) compared to semantic violations (*diaferistis ‘*differ-er’). These studies were later replicated by using stimuli from other, typologically quite distinct languages, such as English (Manouilidou and Stockall, 2014) and Slovenian (Manouilidou et al., 2016). Findings of these later studies are in complete agreement with the original studies conducted in Greek, confirming the architectural model of complex word recognition outlined above.

Moreover, subsequent neuroimaging studies (Neophytou et al., 2018; Stockall et al., 2019) confirm the existence of these two stages and the involvement of syntactic and semantic processing in post-decomposition processes. Specifically, Neophytou et al. (2018), using a subset of the Greek stimuli used in Manouilidou (2007), provided Magnetoencephalography (MEG) evidence for the distinction between these two types of pseudowords which violated syntactic and semantic rules of word formation, in terms of distinct timeframes and brain correlates. In their study, syntactic violations evoked more activity than semantic violations in the temporal lobe in the 200–300 ms time-window, while semantic violations evoked more activity than syntactic violations in the orbitofrontal cortex in the 425–500 ms window. This finding clearly differentiates the two types of information (syntactic vs. semantic) which needs to get processed when dealing with a complex word. This new piece of evidence adds to the growing body of research, which highlights the involvement of various sub-processes during lexical access of complex words and distinguishes *syntactic* vs. *semantic* processing in complex word recognition. Specifically, evidence advocates toward the idea that *syntactic licensing* and *semantic composition* occur at two distinct stages, whereby the former precedes the latter. The same pattern was later replicated in another MEG study by Stockall et al. (2019) by using data from English prefixation. As with Neophytou et al. (2018) and by using the same type of violations, the study targeted the spatial organization and temporal dynamics of morphological processing in the human brain. Results were identical with Neophytou et al. (2018) reinforcing the idea of the existence of stages in lexical processing and their sequence in time.

Thus, taken all this together we have credence to the existence of a consistent architectural map on the complete process of processing morphologically complex words, from initial form-based decomposition to syntactic licensing, and semantic interpretation. Interestingly, combined results of MEG studies from Greek (Neophytou et al., 2018) and English (Stockall et al., 2019) further suggest that the spatial and temporal dynamics of this process are very similar across different languages.

### Morphology in Primary Progressive Aphasia and Stroke-Induced Agrammatic Aphasia

Primary Progressive Aphasia is a neurodegenerative disease which slowly and progressively disrupts the language regions of the brain, resulting in a gradual, and initially isolated, decline in language function (Mesulam, 1982, 2013). Other mental faculties such as memory remain intact, at least at initial stages. According to recent guidelines, PPA can be subdivided into three main variants based on clinical and imaging criteria (Gorno-Tempini et al., 2011; Maruta et al., 2015). What appears to be a common feature is *impaired word knowledge*, mainly manifested as *anomia.* However, there are specific deficits associated with each variant. The main characteristics of the *logopenic variant* (PPA-L) is intermittent word-finding hesitations, impaired phonological memory and problems with repetition (Gorno-Tempini et al., 2011) while their grammatical ability is, in general preserved. However, several recent studies have brought into light various difficulties with grammatical domains, especially in connected speech, such as difficulties with verbal morphology and avoidance of complex structures in production (e.g., Knibb et al., 2009; Ash et al., 2013; Fraser et al., 2014; Marcotte et al., 2017; Mack et al., 2021), albeit these difficulties appear to stem from a general word retrieval and verbal working memory deficit, rather than from an underlying grammatical impairment (Mack et al., 2021). The *agrammatic variant* (PPA-G)^2^ is characterized by impairments of grammar (syntax and morphology) but not of word comprehension. Non-fluent speech, production of grammatically impoverished sentences, verb production difficulties, difficulties with complex syntactic structure (production and comprehension), difficulties in producing function words and bound morphemes, and in general, impaired processing of morphosyntactic structure (e.g., Thompson et al., 1997, 2013; Thompson and Mack, 2014 for a review) also complete the PPA-G profile. Finally, the *semantic variant* (PPA-S) is characterized by impairments of word comprehension, and more specifically by difficulty in processing lexical–semantic information (i.e., word meaning) in both production and comprehension, with associated neural atrophy in the left anterior temporal lobe. Given that no PPA-S patients participated in the study, we will not further elaborate on this condition.

Despite neuropathological differences, similar language deficits, mainly in the syntactic domain, can be found in stroke-induced agrammatic aphasia (StrAg) and in PPA-G as well (Thompson et al., 2012c,^2013^). In particular, the processing of *argument structure* is a domain for which difficulties have been reported for both populations. For instance, difficulties with processing complex argument structure or violations of verb argument structure in narrative speech have been described for both PPA-G and StrAg (Thompson et al., 2012c for PPA-G, Bastiaanse and Jonkers, 1998; Thompson and Bastiaanse, 2012, for StrAg). Thus, it is not uncommon for researchers to compare the two conditions, in order to gain insights about the nature of agrammatism as manifested in two different conditions after brain damage. Even though argument structure difficulties are mostly a result of sentence processing, a comparison of the two groups at the lexical level is valid, given that *inflectional morphology* has been found to be compromised in both PPA-G and StrAg. Interestingly, a recent study by Kordouli et al. (2018) has brought into light interesting dissociations in compound naming between PPA-G and StrAg, with PPA-G performing significantly worse. At the same time, less is known about how patients with PPA and stroke-induced aphasia process *derivational morphology*, which is the topic of the current study.

Derivational morphology, that is the production of a new lexical item from another lexical stem, e.g., *happy* > *unhappy, emerge* > *reemerge* is usually better preserved than inflectional morphology in brain-damaged populations. For instance, Miceli and Caramazza (1988) report on agrammatic aphasic patients’ ability to use derivational affixes as relatively intact. However, subsequent studies brought into light various interesting facts about the processing of derivational morphology by brain-damaged populations. For example, the study by Miceli et al. (2004) reports morphological errors in association with phonological errors, while Faroqi-Shah and Thompson (2010), focusing on complexity as manifested in past tense forms (-*ed*) and progressive aspectual forms (-*ing*), did not find any effects of morphological complexity. On the other hand, Semenza et al. (2002) studied the performance of two Slovenian-speaking patients, one diagnosed with agrammatic aphasia and the other with transcortical motor aphasia. The study showed that while prefixes (e.g., *re-* in *reappear*) are well-preserved in the grammar of both patients, with no phonological distortions on them, at the same time, they can be omitted or substituted. This fact suggests that prefixation, as a morphological operation, and the structure of a prefixed word are preserved in these two types of aphasia. However, the fact that patients do not always succeed in producing the right form of the derived verb suggests certain difficulties with this operation, for both individuals with aphasia (agrammatic and transcortical).

An interesting study by Marangolo et al. (2003) reports on two patients with comparable right hemisphere lesions which involved the gray and white matter of the right temporal and parietal lobes and the right centrum semiovale, who showed a selective deficit in the processing of derived words without any other linguistic deficit. This study was the first one to show that derivational morphology can be selectively impaired and that its processing can be mediated by the right hemisphere. Patients were tested in a picture naming task where they had to name either an action verb or the corresponding derived nouns. They were also asked to produce derived nouns that corresponded to verbs presented to them orally and to produce the verb that corresponded to the nouns they heard. Both patients were unsuccessful in naming derived nouns from verbs (e.g., *liberare* ‘to free’ > *liberazione* ‘freedom’) but they could name verbs from derived nouns (e.g., *liberazione* ‘freedom’ > *liberare* ‘to free’). This study highlights in the best way that derivational morphology can be selectively impaired and that it can have ties with the right hemisphere as well and not necessarily with typical language areas. Finally, not only overt derivation but also zero-derivation (Lukic et al., 2016) appears to be affected in StrAg, especially in cases when aphasic individuals with verb impairments had to “derive” verbs from nouns (*brush* > *to brush*), stressing the crucial role of the grammatical category of the base (i.e., verbs) in performing morphological processes.

Taking all the above into consideration, it appears that derivational morphology leads its own life when it comes to language disorders. On the one hand, it appears better preserved than inflectional morphology. On the other hand, it appears to engage different brain areas, since derived words exhibit a variety of properties that are not found in inflected forms, such as a distinct semantic component, given that the derived word is a separate concept. However, it remains an understudied area in the field of language disorders, thus, calling upon further investigation.

### The Current Study – Research Questions

In the present study we analyze data of complex pseudoword processing from English-speaking individuals diagnosed with two variants of PPA and with StrAg. Given that PPA is a condition which mostly affects lexical processing and given that pseudoword processing touches upon many issues (see Section “Morphological Processing in Healthy Adults”), it appears to be an appropriate domain of investigation in order to see how the underlying deficits of these conditions might affect it. At the same time, a secondary goal is to inform morphological theory by providing independent evidence about a linguistic phenomenon which has occupied the psycholinguistic literature for decades, that is, complex word recognition.

Thus, the overarching aim of the study is to investigate processing of complex pseudowords in these populations and to contribute new data to the literature of lexical/morphological processing by PPA individuals. Within this general frame, we also seek to shed light to related issues, with respect to the type of stimuli investigated and the specific populations that participated in the study. *First* and foremost, given that there is no evidence about complex pseudoword processing, the main aim of the study is to fill this gap of knowledge, thus, making it the first study to bring into light evidence about word-structure building in PPA, a productive operation across languages. *Second*, the specific types of pseudowords used in the current study allow us to investigate the contribution of finer-grained types of information necessary in word-structure building, that is information that pertains to knowledge of the grammatical category of the base and to argument structure specifications. This is particularly important given that no previous study has looked at the influence of both syntactic and semantic properties in the processing of word-building in PPA. Finally, given that both StrAg and PPA-G are characterized by agrammatism, the *third* aim of the study is to compare the two conditions and examine whether agrammatism affects pseudoword processing in the same way.

## Materials and Methods

### Participants

Thirty brain-damaged individuals diagnosed with PPA and meeting the criteria for logopenic (*n* = 12) and agrammatic (*n* = 8) variant or stroke-induced agrammatic aphasia (*n* = 10) were recruited to participate in the study. An additional group of 10 healthy volunteers, aged-matched controls (AM) (5 males and 5 females) were also selected. All participants were monolingual native English speakers with self-reported normal vision and hearing. One healthy AM control was excluded due to poor performance on the lexical decision task, and thus all analyses were done on the remaining 9 AM controls. The participant groups were matched on age [*t*(37) = 1.118, *p* = 0.271] and years of education [*t*(37) = 0.917, *p* = 0.365] although StrAg participants were marginally younger than the participants with PPA [*t*(4) = −2.629, *p* = 0.058].

Individuals with PPA were recruited from the Mesulam Center for Cognitive Neurology and Alzheimer’s Disease in Chicago, IL, United States. All patients were clinically diagnosed with PPA based on neurological examination and related test results [i.e., magnetic resonance imaging and were further categorized by PPA variant based on language and neuropsychological testing, and their magnetic resonance images based on the criteria discussed in Mesulam (2001, 2003, 2013) and Gorno-Tempini et al. (2011)]. Demographics for all participants are presented in Table 1; scores on language measures across participants are provided in Supplementary Appendix 1. None of the PPA patients showed evidence of stroke or other neurological disorder, while all presented a history of progressive language deficits in the face of relatively spared abilities in other cognitive domains. The study was approved by the Institutional Review Board at Northwestern University and informed consent was obtained from all participants.

**TABLE 1:** Participants’ demographic information.

AM	AM01		AM02			AM03			AM04		AM05		AM06		AM07			AM08			AM09		Group average
Age	56		67			60			53		76		64		75			68			63		64.7
Gender	F		F			M			M		F		M		F			M			F		4 M (5 F)
Handedness	R		R			R			R		R		R		R			R			R		all R
Education (years)	18		18			18			18		14		20		21			18			17		18.0

**StrAg**	**SA01**		**SA02**			**SA03**		**SA04**		**SA05**		**SA06**		**SA07**			**SA08**		**SA09**		**SA10**		**Group average**

Age	41		64			29		46		42		22		48			38		67		51		44.8
Gender	M		M			F		M		M		F		M			F		M		M		7 M (3 F)
Months post-stroke	94		13			28		27		20		31		18			98		306		37		67.2
Handedness	R		R			R		R		L		R		R			R		L		R		8 R (2 L)
Education (years)	16		18			19		18		16		14		16			18		20		20		17.5

**PPA participants**	**P1**	**P2**	**P3**	**P4**	**P5**	**P6**	**P7**	**P8**	**PPA-G ave**	**P9**	**P10**	**P11**	**P12**	**P13**	**P14**	**P15**	**P16**	**P17**	**P18**	**P19**	**P20**	**PPA-L ave**	**Total group ave**

PPA-type	**G**	**G**	**G**	**G**	**G**	**G**	**G**	**G**		**L**	**L**	**L**	**L**	**L**	**L**	**L**	**L**	**L**	**L**	**L**	**L**		
Age	76	64	69	74	65	63	66	53	66.25	69	64	80	58	65	70	73	51	60	63	75	67	66.25	66.25
Gender	M	F	F	M	F	M	F	F		F	M	M	F	M	M	M	M	M	M	F	F		11 M (9 F)
Symptom duration (years)	3.6	2.5	2.6	4.9	2.5	7.0	1.5	6.0	3.8	2.6	3.8	6.8	2.0	3.5	2.0	4.0	3.5	4.0	3.5	4.0	4.5	3.7	3.7
Handedness	R	R	R	R	R	R	R	R		R	R	R	R	R	R	R	R	R	R	R	R		20 R
Education (years)	18	16	16	16	14	20	18	16	16.75	16	16	18	19.5	19	17	20	12	18	18	19	18	17.54	17.22

*AM, age-matched; StrAg, stroke agrammatic; SA01, SA02, SA03 etc., stroke agrammatic; PPA, Primary progressive aphasia; G, agrammatic; L, logopenic; S, semantic; P1, P2, P3…etc. Patient; F, female; M, male; R, right-handed.*

The stroke-induced agrammatic aphasic individuals suffered a single left-hemisphere stroke at least 1 year prior to the study with no history of other speech and language impairments prior to stroke. Participants were selected for inclusion based on neuropsychological assessments and according to the criteria of the *Western Aphasia Battery-Revised (WAB-R)* (Kertesz, 2006). Participants exhibited mild-to-moderate aphasia (WAB-AQ mean: 75.4, range: 53.5–89), with non-fluent agrammatic features, such as (a) slow and effortful spontaneous speech (WAB fluency mean: 10.9, range 2–20), (b) impaired comprehension and production of non-canonical sentences, as indicated by performance on the Sentence Comprehension Test (SCT) and the Sentence Production Priming Test (SPPT) of the *Northwestern Assessment of Verbs and Sentences* (NAVS) (Thompson, 2011): for comprehension: non-canonical range: 33.3–86.7% correct; canonical range: 46.7–93.3% correct; for production: non-canonical range: 0–73.3% correct; canonical range: 33.3–100% correct, (c) unimpaired noun production and preserved single-word comprehension of both nouns and verbs, as illustrated by scores ≥ 50% correct on the Confrontation Naming subtest of the *Northwestern Naming Battery* (NNB) (Thompson and Weintraub, 2014; experimental version) and by scores ≥ 60% on the Auditory Comprehension subtest of the NNB, respectively^3^. Details are listed in Supplementary Appendix 1.

### Experimental Conditions and Materials

Four experimental conditions with 40 items each (39 for non-words) and one filler condition were included in the experiment. Specifically, the experimental conditions included one group of non-words (#1 below), two groups of words violating certain constraints of word formation in English (see #2 and #3 below) and one group of real words (#4 below). All were formed with the prefix *re*-. The filler conditions (#5 below) consisted of 80 well-formed words that contained different decomposable affixes (e.g., *unable*). Fillers were used exclusively to distract the participants and to balance the ratio of grammatical vs. ungrammatical words and were not further analyzed. Materials were based on Manouilidou and Stockall (2014). They were modified to comply with the requirements of American English participants i.e., word frequencies of existing items were recalculated based on CELEX English database (Baayen et al., 1995) and a set of new real words were selected. All experimental items were matched for CELEX spoken and written stem/root frequency [for spoken: *F*(3) = 1.095, *p* = 0.353; for written: *F*(3) = 0.049, *p* = 0.986), and for length, apart from real words, which were slightly longer (mean: 7.575, *p* = 0.006 when compared to SynViol and SemViol)]. Finally, durations of auditory files were also calculated. There is no significant difference between durations in the two critical conditions (*t* = −1.056, *p* = 0.297) but they both differ significantly when compared to fillers (*p* = 0.000 in both comparisons). Table 2 presents details on the experimental stimuli.

**TABLE 2:** Characteristics of experimental stimuli.

	SynViol	SemViol	Real	NWs	fillers
**Mean length** **(letters)**	6.85	6.85	7.57	7.35	7.39
**Mean Audio file** **duration (sec)**	0.75	0.77	0.73	0.76	0.66
**Mean stem/root** **frequencies** **(CELEX_log)**	0.98	1.16	1.18		

The stimulus set comprised the following experimental conditions:

(1) Non-words (NWs): pseudowords stems + *re*- (e.g., *repearn; *n* = 39).
(2) SynViol: real word base + *re*-, forming a grammatical category constraint violation (SynViol) (e.g., *resimple; *n* = 40).
(3) SemViol: real word base + *re*-, forming an argument structure/thematic constraint violation (SemViol) (e.g., *rescream; *n* = 40).
(4) Real words with *re*- and no base form violations (e.g., *resubmit*; *n* = 40).
(5) Fillers: real words without *re*- (e.g., *acceptable*; *n* = 80).

In total, the stimuli included 239 words and the ratio between well-formed and ill-formed was 50:50.

### Procedure

An auditory lexical decision task was conducted, running on an IBM computer using E-prime 2.0 professional software (Psychology Software Tools, Pittsburgh, PA, United States), which collected and recorded response time and accuracy data. Initially, participants were given detailed instructions about the experiment and 10 practice trials were provided to familiarize participants with the task. All stimuli were recorded by a native speaker of American English and were presented to the participants *via* headphones. Participants first saw a cross “ +” in the middle of the screen for 1,000 ms and then they heard the stimulus. Participants had to decide as quickly and as accurately as possible whether the word that they heard was a word of English. Participants had 3,000 ms to press with their left hand one of two pre-specified color-coded buttons (either the YES “s” or the NO “a” key), on the left side of the QWERTY keyboard. Participants could pause the task and have a break at any point during the experiment.

## Analysis and Results

A mixed-effects logistic regression was performed on the item-level data for accuracy and a linear mixed-effects regression was performed on the item-level data for reaction times (RT) using the lme4 package in R Studio version 1.2.1335 (Bates et al., 2015; Kuznetsova et al., 2015; R Core Team, 2015; Team, 2018). Participants’ accuracy and the logarithmic transform of their reaction times (logRT) were used as the dependent variables in separate analyses. For both accuracy and reaction time analyses, group (PPA-G, PPA-L, StrAg, and AM), condition (pseudowords with SynViol, pseudowords with SemViol, Non-Words, and real words), and their interaction were entered as fixed factors with age as a covariate, and random intercepts for participants and trial items were entered as crossed random factors in the full model. Models with and without each fixed factor were compared using the anova function in R [see (a) – (e) below for formulas of compared models] to identify the best-fit model for accuracy and RT data separately. In the presence of significant effects, *post hoc* planned comparisons were run, and *p*-values were corrected for multiple comparisons using a single-step method in the multcomp package (Bretz et al., 2010) in R.

Model formulas: For accuracy data, DV = accuracy (1/0), while for RT data, DV = logRT. Formula *d* was the best-fit model for all analyses.

(a) Intercept and random factors only:
    DV ∼ 1 + (1| participant) + (1| item)
(b) Intercept, group, and random factors:
    DV ∼ 1 + group + (1| participant) + (1| item)
(c) Intercept, group, condition, and random factors:
    DV ∼ 1 + group + condition + (1| participant) + (1| item)
(d) Intercept, group, condition, and their interaction, and random factors:
    DV ∼ 1 + group*condition + (1| participant) + (1| item)
(e) Full model: Intercept, group, condition and their interaction, and age (covariate), and random factors:
    DV ∼ 1 + group*condition + age + (1| participant) + (1| item).

With respect to accuracy, group means and standard deviations are presented in Table 3.

**TABLE 3:** Average percent correct (SD) scores for each condition and group.

	SynViol	SemViol	NW	real
StrAg	63 (26.7)^ 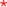 ^	56 (23.3)	76 (23.9)	84 (9.7)
PPA-G	63 (31.0)^ 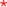 ^	59 (33.2)	78 (17.9)	71 (18.6)^ 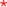 ^
PPA-L	77 (13.1)^ 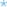 ^	65 (13.2)^ 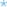 ^	81 (9.8)	79 (14.0)^ 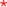 ^
AM	90 (3.3)^ 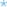 ^	78 (13.1)^ 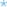 ^	86 (8.4)	92 (5.6)

*Blue asterisk (^
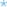
^) indicates significant difference between critical conditions *reheavy vs. *reswim and red asterisk (^
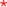
^) indicates significant difference between groups of patients and control group.*

For accuracy data, the best-fit model was the one that included the interaction term [formula (*d*) above; χ^2^(9) = 61.71, *p* < 0.001]. Results from the mixed-effects logistic regression analyses showed a significant group*condition interaction. *Post hoc* comparisons of participant groups indicated that for non-words and real words, the AM group performed better than all patient groups, although this was only significant when comparing the AM group to the PPA groups for real words: (PPA-G: *z* = −3.56, *p* = 0.002; PPA-L: *z* = −2.73, *p* = 0.03). None of the patient groups differed significantly from each other for real words or non-words. Comparisons between groups with respect to the two critical conditions (SynViol and SemViol) revealed the following: For the SynViol condition, the AM group stands out yielding, on average, significantly more accurate rates compared to the PPA-G group (*z* = −2.92, *p* = 0.018) and compared to the StrAg group (*z* = −3.31, *p* = 0.005). None of the patient groups significantly differed from each other for the SynViol condition. For the SemViol condition, the AM group was, on average, only marginally significantly more accurate than the StrAg group (*z* = −2.51, *p* = 0.059). There were no other significant comparisons for the SemViol condition.

*Post hoc* comparisons of conditions for each group indicated no reliable differences between the SynViol and SemViol conditions for the PPA-G group (*z* = −0.82, *p* = 0.85) or for the StrAg group (*z* = −1.75, *p* = 0.30). Interestingly, both the PPA-G and StrAg groups performed significantly better for NWs compared to pseudowords with SynViol (PPA-G: *z* = 4.46, *p* < 0.001; StrAg: *z* = 3.83, *p* < 0.001) and compared to pseudowords with SemViol (PPA-G: *z* = −5.37, *p* < 0.001; StrAg: *z* = −5.81, *p* < 0.001). This suggests that for the two agrammatic groups, the two types of violations (SynViol and SemViol words) are clearly distinguishable from non-words, even though they do not differ between each other. At the same time, both the PPA-L and healthy AM participants produced distinct rates of accuracy for SynViol and SemViol conditions, with significantly better performance for SynViol words (PPA-L: *z* = −3.01, *p* = 0.014; AM: *z* = −3.25, *p* = 0.006). Notably though, the PPA-L group showed no distinct performance between NWs and pseudowords with SynViol (*z* = 1.22, *p* = 0.61).

**FIGURE 1:**
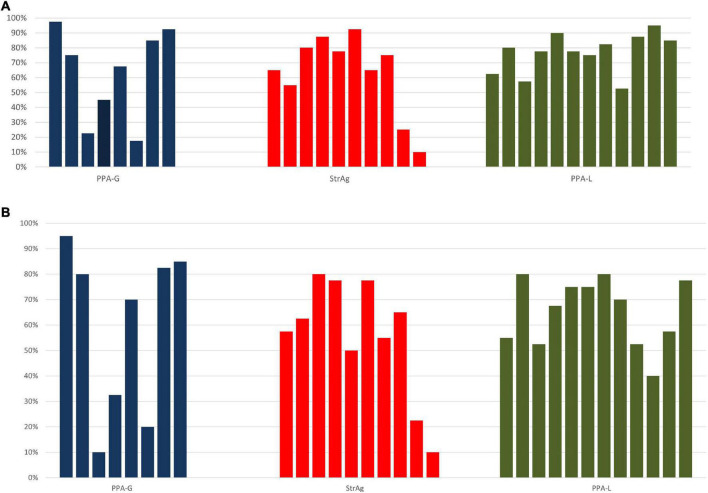
Within group variability across participants [% correct responses for pseudowords with SynViol **(A)** and SemViol **(B)**].

**FIGURE 2:**
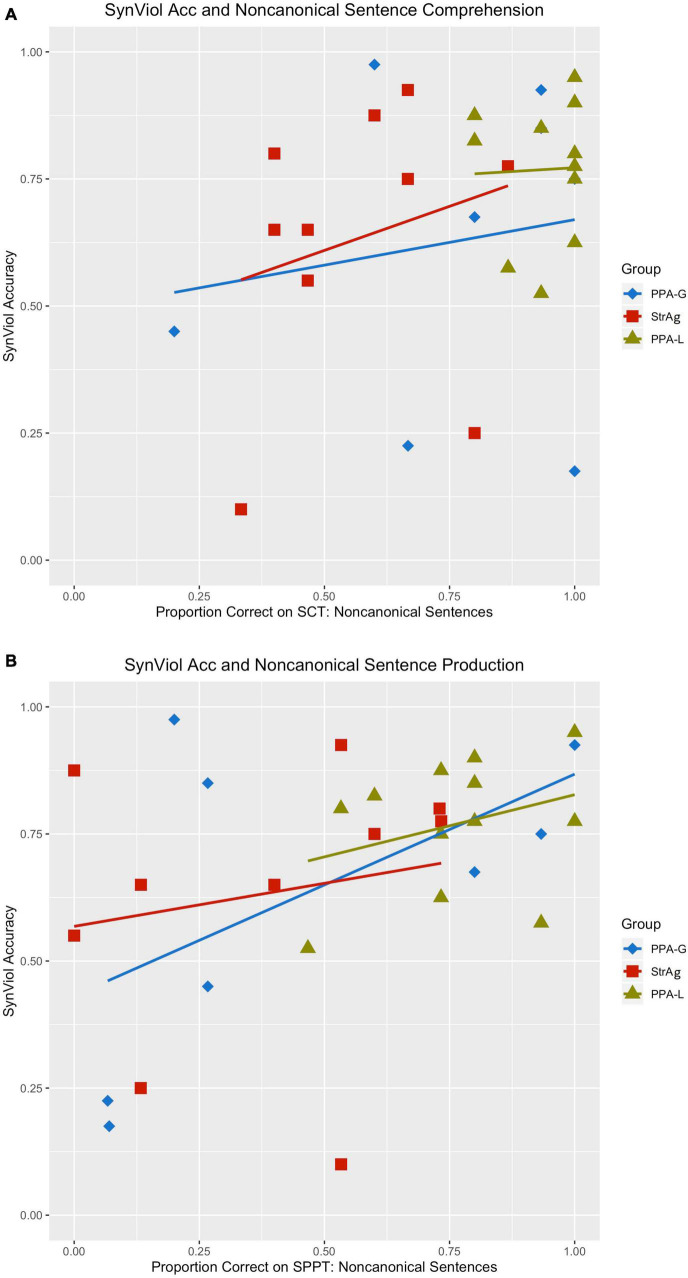
Interaction between accuracy rates for SynViol and language measures as fixed factors. Language measure used: Northwestern Assessment of Verbs and Sentences (NAVS*) Sentence Comprehension Task (SCT) for non-canonical constructions **(A)** and NAVS Sentence Production Priming Task (SPPT) for non-canonical constructions **(B)** (*Thompson, 2011).

Looking at individual responses at Table 4, we see that there is within group variability in the data also illustrated in Figures 1A,B, which is mostly manifested in the two agrammatic groups (for PPA-G, SynViol range: 18–97.5% SemViol range: 10–95%; StrAg, SynViol range: 10–92.5%; SemViol 10–80%). The PPA-L group appears to be less variable (SynViol range: 52.5–90%; SemViol range: 40–80%). Since the PPA classification does not necessarily control for the extent of sentence comprehension/production deficits, we also ran separate models using performance on language measures of non-canonical sentence comprehension (nc*SCT*) and production (nc*SPPT*) instead of group as a fixed factor. These two tasks are not related to the lexical decision task used in the current study, as they tap into participants’ grammatical knowledge, as a broader domain of language knowledge. However, they can provide valuable information with respect to the underlying language deficits of the populations under investigation which can possibly affects participants’ performance in the lexical decision task as well. The same procedure was used to determine the best-fit model using the same formulas listed above (a) – (e), with the only difference of replacing the fixed factor of group with a (continuous) fixed factor for performance on nc*SCT* (percent correct), and separately with a (continuous) fixed factor for performance on nc*SPPT* (percent correct). For the model including sentence comprehension of non-canonical structures (nc*SCT*) as a fixed factor, the best-fit model was the one that included the interaction term [χ^2^(3) = 35.84, *p* < 0.001]. Results from the logistic regression analysis showed a significant interaction between condition and performance on nc*SCT. Post hoc* comparisons revealed that performance on nc*SCT* was not a significant predictor of accuracy for any of the conditions. For the model including sentence production of non-canonical structures (nc*SPPT*) as a fixed factor, the best-fit model was the one that included the interaction term [χ^2^(3) = 50.73, *p* < 0.001]. Results from the logistic regression analysis showed a significant interaction between condition and performance on nc*SPPT. Post hoc* comparisons indicated that the *ncSPPT* language measure was a significant predictor of SynViol accuracy (*z* = 2.52, *p* = 0.01), but not of accuracy for the other conditions. As shown in Figure 2, the degree of impairment of grammatical abilities agrees with accuracy in detecting SynViol, while no significant interactions were found for SemViol.

**TABLE 4:** Individual responses (% correct responses) per experimental condition.

SynViol accuracy	P1	P2	P3	P4	P5	P6	P7	P8	P9	P10	P11	P12
StrAg	65%	55%	80%	87.50%	78%	92.50%	65%	75%	25%	10%		
PPA-G	97.50%	75%	23%	45%	68%	18%	85%	93%				
PPA-L	62.50%	80%	57.50%	77.50%	90%	78%	75%	83%	52.50%	88%	95%	85%

**SemViol accuracy**	**P1**	**P2**	**P3**	**P4**	**P5**	**P6**	**P7**	**P8**	**P9**	**P10**	**P11**	**P12**

StrAg	57.50%	62.50%	80%	77.50%	50%	77.50%	55%	65%	22.50%	10%		
PPA-G	95%	80%	10%	32.50%	70%	20%	82.50%	85%				
PPA-L	55%	80%	52.50%	67.50%	75%	75%	80%	70%	52.50%	40%	57.50%	77.50%

Comparisons between groups revealed that the PPA-L group performed significantly better than the StrAg group for these two language measures (nc*SCT*: *z* = −4.86, *p* < 0.001; ncSPPT: *z* = −3.16, *p* = 0.005) and significantly better than the PPA-G group for nc*SPPT* (*z* = 2.41, *p* = 0.04). The StrAg group performed worse than the PPA-G group for nc*SCT* (marginal significance: *z* = −2.32, *p* = 0.053), but not for ncSPPT (*z* = −0.53, *p* = 0.86; see Table 5 for SCT and Table 6 for SPPT). Participants’ percentages of correct responses in these tasks can be found in Supplementary Appendix 1, the relevant part repeated here for convenience in Table 7.

**TABLE 5:** Between group comparisons for SCT.

Comparison	Estimate	Standard error	*Z*-value	*P*-value
PPAL – PPAG	0.178	0.083	2.15	0.081
StrAg – PPAG	–0.200	0.086	–2.32	0.053
StrAg – PPAL	–0.378	0.078	–4.86	< 0.001***

Significance level: *p < 0.05; ^**^p < 0.01; ^***^p < 0.001.

**TABLE 6:** Between group comparisons for SPPT.

Comparison	Estimate	Standard error	*Z*-value	*P*-value
PPAL – PPAG	0.311	0.129	2.41	0.042*
StrAg – PPAG	–0.071	0.134	–0.53	0.86
StrAg – PPAL	–0.381	0.121	–3.16	0.005**

Significance level: *p < 0.05; ^**^p < 0.01; ^***^p < 0.001.

**TABLE 7:** Percentages of correct responses (standard deviations) per group for language measures of comprehension (SCT) and production (SPPT) of non-canonical sentence structures.

	SCT	SPPT
**StrAg**	56.7 (18.12)	38.0 (28.94)
**PPA-G**	76.7 (27.36)	45.0 (39.26)
**PPA-L**	94.4 (7.95)	76.1 (16.92)

With respect to RTs, only response latencies corresponding to correct trials were analyzed, and RTs smaller than 300ms were eliminated for all participants (less than 1% of the data). Although the model was run using log-transformed data, raw values are presented in Table 8 for easier interpretability. For RT data, the best-fit model was the one that included the interaction term [formula (*d*) above; χ^2^(9) = 59.86, *p* < 0.001]. Results from the linear mixed-effects regression analyses showed a significant group*condition interaction. *Post hoc* comparisons of participant groups indicated no significant differences for SynViol or SemViol words. *Post hoc* comparisons of conditions for each participant group revealed that all groups performed faster for both NW and real words compared to the critical conditions (SynViol and SemViol) (*p* < 0.001)^4^, but only the AM group differentiated between the two critical types of violation (syntactic and semantic) by presenting significantly faster responses in the former type of violation (*z* = 3.279, *p* = 0.005). We also modeled for interactions with performance on language measures (*ncSCT*/*ncSPPT*), but there were no significant outcomes.

**TABLE 8:** Mean RTs (SD) in milliseconds for each condition and group for only correct responses > 300 ms.

	SynViol	SemViol	NW	real
StrAg	1379 (270)	1416 (270)	1279 (268)	1280 (208)
PPA-G	1488 (218)	1551 (217)	1420 (220)	1340 (182)
PPA-L	1596 (173)^ 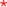 ^	1636 (143)^ 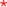 ^	1463 (150)	1415 (123)
AM	1389 (105)^ 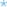 ^	1477 (103)^ 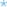 ^	1320 (97)	1159 (73)

*Blue asterisk (^
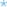
^) indicates significant difference between critical conditions *reheavy vs. reswim and red asterisk (^
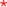
^) indicates significant difference between groups of patients and control group.*

In sum, the current pattern of results can be summarized as follows. Healthy controls distinguished the two critical conditions, both in terms of accuracy and RTs, with SynViol being easier and faster to reject compared to SemViol. With respect to patient groups, closer to AM was the PPA-L group, as they were the only group which did tell apart the two critical conditions based on error rates, however, RTs did not indicate distinct timeframes in terms of processing. PPA-G and StrAg were comparable to each other, not being able to tell apart the two critical conditions, but clearly isolating them from both real words and non-words. Finally, participants’ performance on *ncSPPT* was a strong predictor for their accuracy rates on SynViol. Based on this summary, we will discuss our data in the following section.

## Discussion

The current investigation aimed at: (a) examining the ability of PPA and StrAg individuals to process pseudowords and more specifically to detect violations in deverbal word formation, (b) isolating the contribution of each type of relevant information (e.g., syntactic vs. semantic) in deverbal word structure building and (c) comparing the performance of PPA-G and StrAg, two conditions characterized by agrammatism, in order to detect its effect in pseudoword processing. For the investigation of the above questions, we will focus on the data obtained for the two critical conditions, that is SynViol and SemViol, and we will consider participants’ scores on both *accuracy* of response as well as *reaction times.*

Looking at accuracy data, when it comes to the first question, the PPA-L group appears to be the group with the best performance, as it does not differ significantly from AM in either the detection of SynViol or SemViol. The two agrammatic groups clearly have difficulties with the detection of SynViol, as they both differ significantly from AM, while for SemViol, the PPA-G group did not differ from AM while there was only a marginally significant difference between StrAg and AM^5^. This is a first indication that the two agrammatic groups have an increased difficulty in accessing information of syntactic nature within a complex word. A comparison among experimental conditions within groups reveals further important dissociations. Addressing the second question will shed more light into the source of these differences.

The second question aimed at investigating whether the groups of participants can process separately the two types of information (syntactic vs. semantic) in pseudoword lexical access. In other words, we seek to examine whether they can tell apart the two critical conditions which will also help us further investigate the source of their difficulties. For this purpose, we are looking for distinct accuracy rates for SynViol and SemViol across participant groups. It seems that while all participants had higher accuracy rates for SynViol, this difference reached significance only for PPA-L but not for the two agrammatic groups, PPA-G and StrAg, which appear to treat them alike (*resmile = *rehappy). If this is the case, then we would have to assume that the PPA-L group is able to process separately information associated with the grammatical category of the base and information associated with argument structure. The group of PPA-L performed as expected, that is, they processed the two types of information, and they did not differ from AM controls, suggesting a better preserved morphological and lexical system than the two agrammatic groups.

Finally, there is a dichotomy between the two agrammatic groups (PPA-G and StrAg) and the PPA-L group. PPA-G and StrAg groups did not differ significantly, neither with respect to overall accuracy rates nor with respect to accuracy rates regarding the two types of violations. This lack of difference is in line with previous studies comparing the two conditions in various grammatical tasks and it suggests a unified effect of agrammatism in detecting word-formation violations. Of particular interest is the performance of these groups when it comes to language measures which target the investigation of their grammatical abilities (see Figure 1). Specifically, grammatical abilities (as modeled through complex sentence production), turned out to be a significant predictor for SynViol accuracy. Several studies have shown that individuals with acquired aphasia often present with sentence comprehension and production deficits in sentences with non-canonical word order, such as passives and object relative clauses, compared to those with a basic, canonical Subject, Verb, Object (SVO) order (for production: Schwartz et al., 1994; Friedmann and Grodzinsky, 1997; Caplan and Hanna, 1998; for comprehension: Caramazza and Zurif, 1976; Schwartz et al., 1980; Caplan and Futter, 1986; Grodzinsky and Finkel, 1998; Friedmann and Shapiro, 2003; Thompson and Shapiro, 2005). Thus, what unites these two groups is their compromised grammatical knowledge, which appears to be a decisive factor for the detection of SynViol. Similarly, while both populations have clear and well-documented difficulties with identifying violations of verb argument structure at the sentence level (Thompson et al., 2012c), the image, as emerged in the current study, is not that clear at the lexical level as there is no statistical difference with the control group when detecting SemViol, that is argument structure violations at the lexical level. If further research establishes this finding, it could suggest that the source of their sentence deficit does not have to do with a loss of argument structure knowledge but with a difficulty processing it at the sentence level, as also suggested in Thompson and Mack (2019).

Results on RTs add a different dimension to the current investigation. First, results of AM controls were in line with previous studies dealing with these types of violations. That is, healthy participants produced distinct RTs for each type of unattested pseudowords, and most importantly, they distinguished SynViol from SemViol. This piece of evidence suggests that speakers are selectively sensitive to levels of linguistic analysis when it comes to lexical processing. This on its own is an important piece of information. However, the interesting issue to be addressed is what this selective sensitivity reflects. It can reflect a qualitative difference between the types of information one needs to evaluate during lexical processing, suggesting an “ease” of detecting a violation of syntactic type in word formation. That is, speakers need more time to evaluate semantic information compared to syntactic information. This is a plausible interpretation, given the nature of the pseudowords used in the current study, as the processor seeks interpretable situations for SemViol, and this “search” could be easily reflected in RTs. On the other hand, the observed pattern might also reflect a deeper architectural mechanism in word-building structure. We will discuss this possibility in the following paragraphs.

This pattern further supports the argument put forward by Manouilidou (2006, 2007), Manouilidou and Stockall (2014), and further validated by Neophytou et al. (2018) and Stockall et al. (2019), that the processing of the grammatical category information temporally precedes the processing of the argument structure information. Looking at the broader picture, these results support the idea that syntactic licensing and semantic composition occur at two distinct stages, the former preceding the latter. With respect to our first question, the two types of violations did not produce distinct RTs for any group of pathological populations. In contrast, RT patterns obtained from PPA-L, PPA-G and StrAg suggest that participants from these groups did not process these two types of critical stimuli at distinct timeframes. This could mean that their overall approach to these types of pseudowords was not to process them at distinct stages but altogether, in a more holistic way. However, they all process them at distinct timeframes compared to NWs and real words, suggesting that for each group SynViol and SemViol are not pure NWs, and that participants tried to interpret them but failed to tell them apart.

Taken together, the results from accuracy and RTs as well as our previous knowledge about the processing for these pseudowords by healthy participants, one can make the following observations. Let us assume a staged lexical access, as outlined in Schreuder and Baayen (1995), Burani et al. (1999) and Fruchter and Marantz (2015). At an initial stage, decomposition occurs, and all lexicalized substrings are exposed. This is when NWs (*repearn) are processed and rejected as bearers of a non-existent stem. The second stage is where syntactic licensing occurs, and the stage during which SynViol (*recomplex) are dealt with. The third stage is dedicated to semantic processing or recombination, and it is the stage where SemViol (*reswear) are processed.

The AM controls follow this pattern as reflected in distinct RTs produced for each category. The lack of difference at the RTs between SynViol and SemViol for all patient groups is suggestive of the following scenarios which should be considered with caution, given the variability among our participants in pathological groups and the confounding effect it might have. First, either stages 2 and 3 are unified as one stage (where both syntactic and semantic information are being processed) or one of them (either syntactic or semantic) is eliminated or skipped depending on the deficit of the specific population. On the grounds of this, let us examine the performance of all groups of participants. Accuracy rates suggest that the two agrammatic groups (PPA-G and StrAg) do not distinguish between SynViol and SemViol. Thus, the first thought would be to assume that agrammatic speakers have one single stage (a combination of stages 2 and 3) where any kind of information is being processed. However, given that the reduced grammatical abilities of these two groups are a strong predictor for accuracy rates, it is plausible to assume that what they miss is the “hardware” to perform syntactic licensing (stage 2), thus judging pseudowords with violations only at the semantic level, where both SynViol and SemViol fail to pass. This pattern explains both the lack of distinct RTs and the lack of distinct accuracy rates for these two groups.

On the other hand, accuracy rates suggest that PPA-L distinguish SynViol from SemViol. Thus, they must have access to the different kinds of information that are violated in each formation. The PPA-L group yielded the highest accuracy rates, and it was the only group which did not differ from controls. It is the group that demonstrates the most consistent (smallest variability) and best-preserved performance when it comes to detecting violations and for telling them apart. This is in accordance with their profile as demonstrated in the literature (Thompson et al., 2012a; Thompson and Mack, 2014). That is, while derivational morphology has not been examined in PPA-L, evidence from inflectional morphology suggests that patients do not have difficulties in the production of morphology. In other words, their performance in processing pseudowords is compatible with their manifested lexical difficulties stemming mostly from the phonological component of lexical knowledge (Mack et al., 2013, 2021), a deficit that could not have interfered with the nature of a lexical decision task. However, their high RTs (overall significantly slower than StrAg and AM) suggest a processing slowdown which could also be responsible for the lack of RT difference between the critical conditions (SynViol vs. SemViol), possibly as a speed-to-accuracy trade-off^6^.

Before we conclude anything along the previous lines about PPA-L, an important piece of information that we should consider is the fact that accuracy rates for SynViol do not differ from NWs in this group. This suggests a robust rejection of these formations as pure non-words, possibly by applying a coarse structural well-formedness criterion, rejecting them without hesitation and being unsure about finer-grained distinctions such as SemViol. Even though semantic impairments are not the main feature of PPA-L, there have been studies in the literature, suggesting faulty semantic processing as well (Rogalski et al., 2008; Thompson et al., 2012b; Barbieri et al., 2021). Specifically, in Barbieri et al. (2021), individuals with PPA-L failed to detect violations of argument structure (which constitute the basis of our SemViol) in an EEG sentence processing experiment. Hence, one could claim that the difference between the two (SynViol and SemViol) appears to stem from a sensitivity to what is being violated at the syntactic level and a slight disturbance at the semantic level. Thus, it seems that there is a dichotomy between the two agrammatic groups on the one hand and the PPA-L group on the other hand, with the first ones judging the pseudowords under investigation at a semantic level and the latter ones, judging them at a structural well-formedness level. In fact, such a dichotomy, agrammatic groups on the one hand and PPA-L on the other, has already been manifested in previous studies (Thompson and Mack, 2014) examining grammatical impairments in all variants of PPA.

Thus, if we indeed accept a staged lexical access as outlined in Section “Morphological Processing in Healthy Adults,” we will have to assume a two-way performance for our groups of participants. Specifically, agrammatic groups fail to fully apply the syntactic licensing criterion – they judge them at the semantic level (different from AM when it comes to SynViol), a judgment which produces similar accuracy at similar timeframes for both SynViol and SemViol. Ultimately, the two agrammatic groups are not selectively sensitive to various levels of linguistic analysis, as they treat both violations as semantic. Finally, PPA-L demonstrates performance with the highest accuracy rates (like AM controls), an indication of a preserved ability to process morphologically complex words, albeit with the slight interference of a possible semantic disturbance [as in Barbieri et al. (2021)].

Finally, we will conclude this section with a comment on the issue of variability. Variability among participants has been a feature of many pathological conditions and it is very well manifested in aphasia. It has also been one of the methodological challenges in group studies. Genuine individual differences exist in every aspect of human existence. It is the challenge for the researcher to pin down their source, to the extent that this is possible. In our study, within group variability is undeniable and it is mostly manifested within the two agrammatic groups. However, when controlling for this individual variation by using participants as a random factor in our mixed models, a uniform pattern emerges, and it is in accordance with the patients’ clinical and cognitive profile. Furthermore, by modeling for language measures, we have shown how variation (in dealing with these pseudowords that deviate from canonicity) can be understood and we have identified its source.

## General Discussion and Conclusion

Language research on brain-damaged populations is informative for two main reasons; first it contributes to the understanding of the pathology; second it allows us to learn more about the normal process. Before we conclude, we will address these two points having in mind the findings of the present study.

The hallmark of PPA is impaired *word knowledge*. Given the vastness of this, the current study is the first one attempting to shed light onto finer aspects of word knowledge in PPA, by using a linguistically informed approach in order to provide detailed profiles of linguistic strengths and weaknesses of the populations under investigation. We focused on complex pseudowords, aiming at investigating morphological processing, an under-studied domain when it comes to language disorders. As outlined in the introduction, morphological processing requires the combination of knowledge of various linguistic domains, such as syntax and semantics. With this in mind, we aimed at examining how the specifics of each PPA variant under consideration could be affected.

This study allowed us to confirm some facts about the different variants and it also brought into light new insights. First, the study provides evidence for a unified effect of agrammatism, resulting from stroke and from a neurodegenerative disease, at the lexical level. What we knew up until now is that the two populations demonstrate similar performance at the sentence level and in syntactic tasks (Thompson et al., 2012c; Thompson and Mack, 2014). The current study brought into light striking similarities at the lexical level as well suggesting that both groups operate in the same way when judging pseudowords as well. Given that their performance correlates with their weak grammatical abilities altogether, we have evidence that they rely on their semantic knowledge rather than on anything else in order to process these pseudowords.

The current dataset also brought into light a dichotomy between the two agrammatic groups and PPA-L, as it is also reported in Thompson and Mack (2014). Results are in line with the profiles of PPA-L, as manifested in the literature, that is, a relatively good performance of PPA-L at detecting violations at the lexical level (no difference compared to the AM group). Given the scarcity of chronometrized studies when it comes to PPA, what we did not know before is that PPA-L shows a speed-accuracy trade-off effect, suggestive of their strategy in dealing with these pseudowords. In other words, this group approaches with caution the lexical decision task, taking time in using their relatively preserved abilities.

Overall, the novelty of the current study with respect to PPA is that it provides an explanation for what “impaired word knowledge” could mean by revealing the different strategies of these populations when confronted with pseudowords, thus allowing a window to our understanding on how these populations treat any complex lexical item. Therefore, when we say that PPA affects word knowledge, the current study offers an account as to what might be the underlying reason for failing word knowledge for the variants under consideration.

Looking at the other side of the coin, the present study offered an alternative way of looking at morphological operations. Most psycholinguistic literature postulates the existence of various stages in accessing complex pseudowords, each stage being devoted to the processing of specific types of information. The present study confirms this procedure, albeit in an alternative way. The lack of time differences in the processing of SynViol vs. SemViol does not allow us to clearly talk about temporal stages. However, combined results from RTs and accuracy confirm the different types of information that are involved in these types of structures.

First, looking at the performance of PPA-G and StrAg when it comes to SynViol and the fact that this performance is predicted by their weak grammatical abilities altogether, we have a first-hand piece of evidence that grammatical knowledge is at stake when it comes to processing these pseudowords. Alternatively seen, syntactic licensing is an obligatory step in complex word recognition, a step which is being compromised by agrammatism. Taken together with their control-like performance for the SemViol condition, we have the second piece of evidence that although SemViol words result from violating argument structure specifications, they are ultimately processed at a semantic level, as semantic recomposition suggests (Fruchter and Marantz, 2015). This distinction between the types of information being processed is further reinforced by the performance of the PPA-L group.

Thus, the current study evidently and inevitably provides further input to our knowledge about morphological processing of complex words in a totally innovative way. Empirical evidence of this type constitutes a contribution to our perception of morphology which is beyond the theoretical level. Given the increase of linguistically informed research in language disorders, the role of this type of study to our understanding of normal language may turn out to be vital, in a way that, until recently, might have looked unimaginable.

## Data Availability Statement

The original contributions presented in the study are included in the article/Supplementary Material, further inquiries can be directed to the corresponding author.

## Ethics Statement

The studies involving human participants were reviewed and approved by Institutional Review Board (IRB) of Northwestern University. The patients/participants provided their written informed consent to participate in this study.

## Author Contributions

All authors listed have made a substantial, direct, and intellectual contribution to the work, and approved it for publication.

## Conflict of Interest

The authors declare that the research was conducted in the absence of any commercial or financial relationships that could be construed as a potential conflict of interest.

## Publisher’s Note

All claims expressed in this article are solely those of the authors and do not necessarily represent those of their affiliated organizations, or those of the publisher, the editors and the reviewers. Any product that may be evaluated in this article, or claim that may be made by its manufacturer, is not guaranteed or endorsed by the publisher.

## Footnotes

1 Schreuder and Baayen (1995) acknowledge the complications of auditory lexical access when it comes to segmentation and mapping of speech input on form-based access representations. “*Prosodic information, resyllabification, stress shifts, tone sandhi, and other phonological mutations may complicate this mapping operation*” (p. 133).

2 In some patients, grammar and comprehension are jointly impaired early in the disease. These patients can be said to have a fourth ‘*mixed’ variant* (PPA-m) (Mesulam, 2013).

3 NNB scores are missing for one participant, SA09, due to time constraints when testing.

4 For PPA-G the comparison between NW vs. SynViol was only marginally significant (*p* = 0.0535) and for the AM group, *p* = 0.0162 for NW vs. SynViol.

5 Following Olsson-Collentine et al. (2019), we will interpret this difference as “insignificant.” According to the study values between 0.05 and 0.10 are known to have low evidential value and they should be treated as insignificant.

6 Similar speed-to-accuracy trade-off effects were also reported for a combined PPA-L/PPA-G group in a word comprehension eye-tracking study (Seckin et al., 2016).
