# Supplementary material for: What Language Disorders Reveal About the Mechanisms of Morphological Processing
Source: Front Psychol. 2021 Nov 29;12:701802. doi: 10.3389/fpsyg.2021.701802 (PMC8667867; doi:10.3389/fpsyg.2021.701802)
Supplement: Supplementary file 1 [file Table_1.docx]

Appendix

Participants’ language profile.


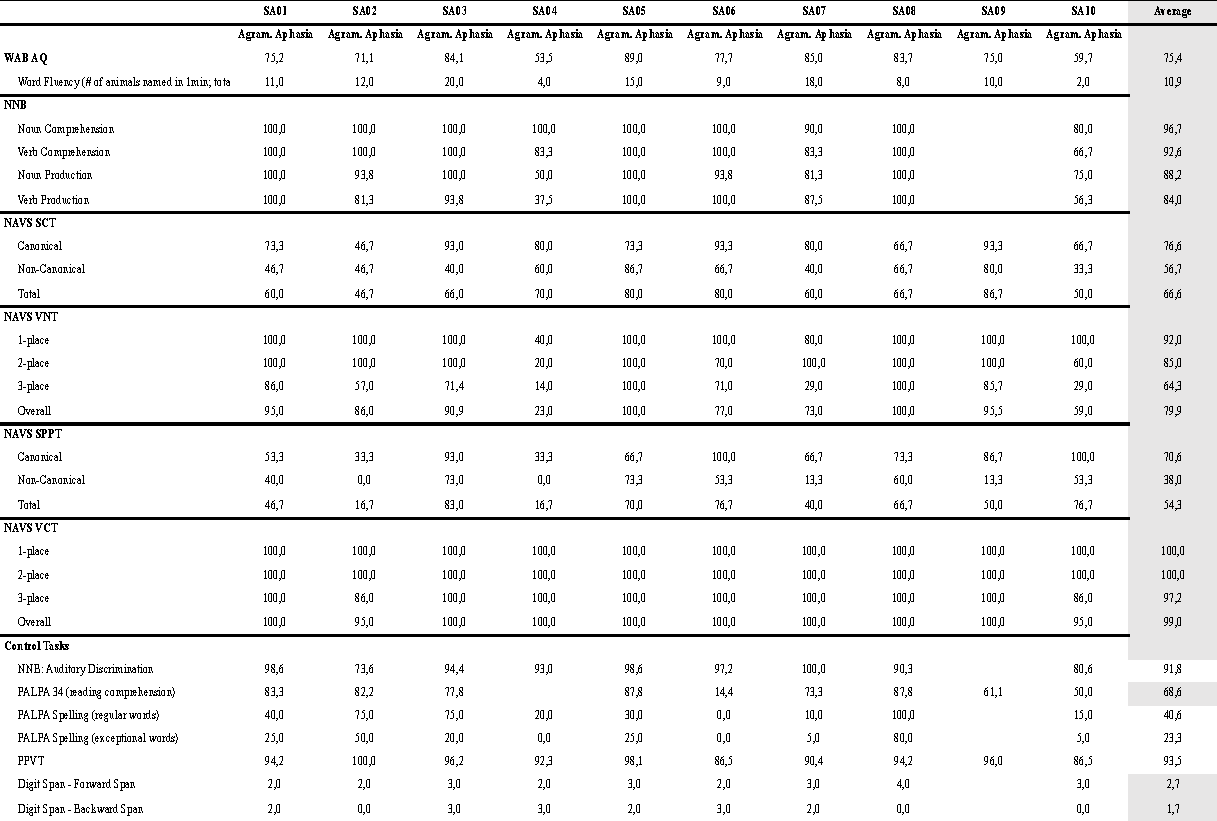


Table 1(a). Language measures for agrammatic participants.


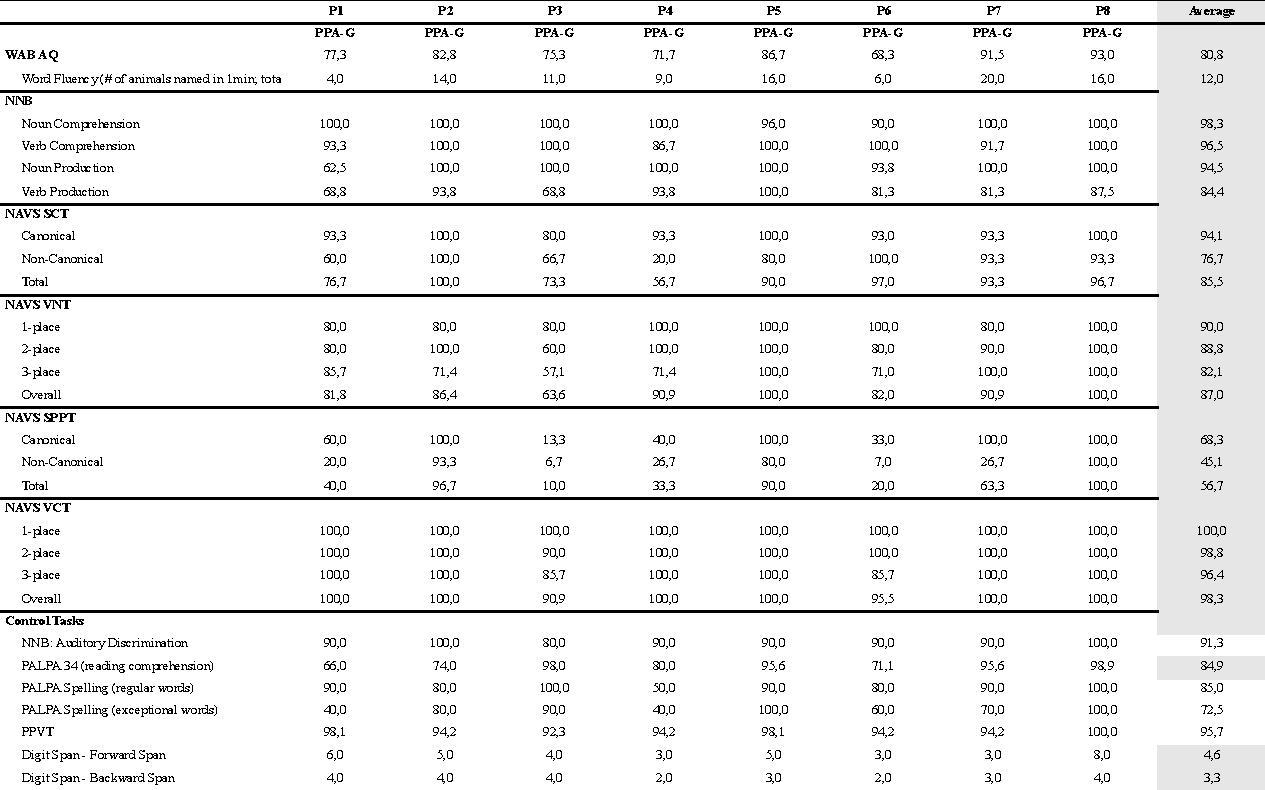


Table 2(b). Language measures for PPA-G participants.


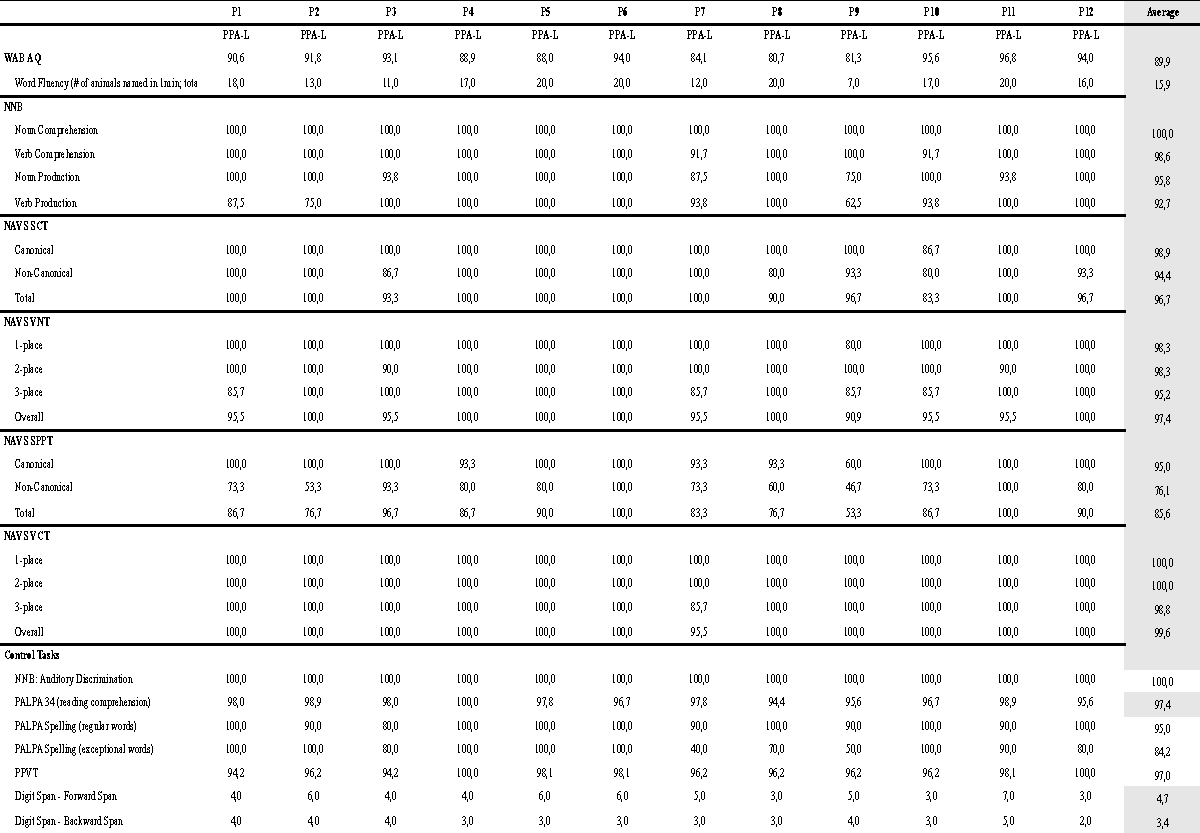


Table 3(c). Language measures for PPA-L participants.
